# Work Ability in Patients with Chronic Myeloid Leukemia: A Danish Nationwide Cohort Study

**DOI:** 10.3390/cancers17091585

**Published:** 2025-05-07

**Authors:** Eva Futtrup Maksten, Jonas Faartoft Jensen, Gitte Thomsen, Ditte Rechter Zenas, Maren Poulsgaard Jørgensen, Lene Udby, Kirsten Fonager, Marianne Tang Severinsen

**Affiliations:** 1 Department of Haematology, Clinical Cancer Research Center, Aalborg University Hospital, 9000 Aalborg, Denmark; j.faartoft@rn.dk (J.F.J.); gith@rn.dk (G.T.); maren.joergensen@rn.dk (M.P.J.); m.severinsen@rn.dk (M.T.S.); 2 Department of Clinical Medicine, Aalborg University, 9000 Aalborg, Denmark; k.fonager@rn.dk; 3 Department of Haematology, Zealand University Hospital, 4000 Roskilde, Denmark; lud@regionsjaelland.dk; 4 Department of Social Medicine, Aalborg University Hospital, 9000 Aalborg, Denmark

**Keywords:** chronic myeloid leukemia, work disability, cancer treatment adverse events, quality of life in cancer patients

## Abstract

**Background/Objectives**: Patients with chronic myeloid leukemia (CML) have a long life expectancy due to modern treatment. However, treatment may have adverse effects that hamper work ability. **Methods**: Patients aged 25–60 years diagnosed in 2002–2020 were included in this nationwide matched cohort study examining work ability from diagnosis (index date), including the need for permanent disability compensation (flexible job or disability pension). Each patient was matched 1:5 on sex, birth year, and level of comorbidity with citizens from the general Danish population without CML. The risks of requiring flexible job and disability pension were calculated by cause-specific hazard ratios (HRs) using Cox proportional hazards regression, and the Aalen–Johansen estimator was used to determine cumulative risks. **Results**: A total of 489 patients with CML and 2445 matched comparators were included. The median age was 46 years, and males comprised 59.5% of the cohort. Matched comparators were more likely to work at index date and 1, 3, 5, and 10 years after the index date (*p* < 0.001). The HRs of requiring both flexible job (HR 8.7 (95% confidence interval (CI): 6.1;12.2, *p* < 0.001)) and disability pension (HR 3.7 (95% CI: 2.8;4.9, *p* < 0.001)) were higher among patients diagnosed with CML compared to matched comparators. The cumulative risk of requiring flexible job and disability pension also increased in patients with CML compared to matched comparators (*p* < 0.001). **Conclusions**: Patients with CML have a reduced work ability compared to the general population. More research is needed to determine the cause of their loss of ability to work.

## Simple Summary

Patients with chronic myeloid leukemia (CML) often live as long as their peers due to treatment. However, the treatment has side effects that can interfere with work ability. The aim of this study was to determine work ability among patients of working age diagnosed with CML. This was established using the Danish registers. Patients with CML were compared with their peers without CML of same age and sex. A total of 489 patients with CML and 2445 peers were included. The peers were more likely to hold a job up to 10 years after the time of diagnosis. Moreover, patients with CML needed permanent disability compensation due to loss of work ability more often. The findings are important as work ability has a high impact on the quality of life for many persons, including patients with cancer.

## 1. Introduction

Chronic myeloid leukemia (CML) is a chronic Philadelphia-positive myeloid neoplasia which saw a dramatic change in survival after the introduction of the first Tyrosine Kinase Inhibitor (TKI) in 2001. The follow-ups of participants in clinical trials show a 10-year survival now exceeding 80% [1,2]. After a few months of treatment, most patients attain a normalization of bone marrow function, and all signs of the cancer vanish within the bone marrow. Patients are therefore often expected to live a normal life in every context.

However, the treatment often comes with adverse effects. Musculoskeletal pain, fatigue, nausea, diarrhea, and headache are common long-term adverse effects of TKI treatment. Both CML itself at the time of diagnosis and the later adverse effects may affect the patients’ ability to work. Ability to work is highly relevant in patients with CML, as it is a significant factor for quality of life [3]. An earlier study has shown that patients with CML are among the patients with hematological cancer that have the highest rate of return to work after diagnosis, but information on their work ability after the initial return to work is unknown [4]. Moreover, compared to other patients with hematological cancer, the proportion of patients with CML who have a permanently reduced work ability, which induces the granting of work disability compensation, is high [5,6]. Two recent French studies have shown that 65% of patients with CML are in need of work modifications, and more than 40% have at least one period of sick leave within the first two years after treatment initiation [7,8]. But there is a lack of studies on long-term work ability in patients with CML within the TKI era. Earlier studies have only included short-term follow-ups [7,8] or included patients diagnosed within the pre-TKI era [4,5,6].

As the Danish welfare system allows for compensation in case of reduced work ability, it is a unique setting for evaluating work function after the diagnosis of CML. The aim of the present study was to assess long-term work ability among patients diagnosed with CML in the TKI era and to assess the need for permanent work disability compensation compared to matched comparators without CML from the Danish population.

## 2. Materials and Methods

### 2.1. Study Population

Patients aged 25 to 60 years with a first-time diagnosis of CML between 1 January 2002 and 31 December 2020 were included in this study. Patients were identified through the Danish National Pathology Registry and the Danish National Chronic Myeloid Neoplasia Registry using the Danish pathology codes (the Danish version of the Systematized Nomenclature of Medicine (SNOMED) codes M98633 and M98753 [9,10]). The date of diagnosis was defined as the index date.

Patients granted any pension (disability, early retirement, or retirement) or flexible job (granted to persons with permanent partly reduced work ability) before the diagnosis of CML were excluded. Furthermore, patients not living in Denmark at the time of the diagnosis were excluded.

A cohort of matched comparators without CML was identified with individuals drawn from the Danish Civil Registration System (CRS) [11,12]. Each patient was matched randomly without replacement with five comparators using sex, birth year, and level of comorbidity as matching variables. The level of comorbidity was calculated 180 days before the date of diagnosis (or a corresponding date for matched comparators) using a modified Charlson comorbidity index (CCI) excluding leukemia [13,14,15]. The CCI was calculated based on information from the Danish National Patient Register and supplemented with information from the National Prescription Registry (diabetes treated by general practitioners) and the Danish Psychiatric Central Research Register (dementia diagnosed by psychiatrists) [16,17,18,19,20]. All matched comparators were living in Denmark at the index date (the corresponding patient’s date of diagnosis) and were not previously diagnosed with CML or granted flexible job or any pension (disability, early retirement, retirement) before the index date.

### 2.2. Outcomes and Confounders

The Danish welfare system ensures economic support when a citizen is either temporarily or permanently unable to work. In case of temporary sick leave, the citizen is paid their normal salary by the employer, which after four weeks is partly reimbursed by the state. In the case of unemployment, the sickness benefit is paid directly to the citizen by the state. If the work ability is permanently reduced, the citizen can either be granted flexible job or disability pension. Flexible job is granted if work ability is partly lost. In this case, work hours and obligations are scheduled according to the citizen’s work ability with salary compensation for the discrepancy between the actual worked hours and a normal full-time work from the state. If the work ability is completely lost, the citizen can be granted disability pension with full compensation from the state. Data on social payments including sick leave and pensions are available and updated weekly in the DREAM registry (Danish acronym for “The evaluation of the extent of the marginalisation based on registries”) [21]. All payment codes in DREAM were categorized into working, unemployed, sick leave, flexible job, disability pension, early retirement pension, retirement pension, or unclassified (Supplemental Table S1). For the sub-analysis looking at work ability state proportions over time from the index date, early retirement and retirement pension were combined into one to avoid personally identifiable data due to small groups. Patients and matched comparators were excluded from this sub-analysis at the time of censoring or death.

The first date of the first week with a social payment for disability pension, early retirement pension, retirement pension, or flexible job was used as the date of the respective type of social payment. The employment status before the index date was calculated as the predominant status from week thirty-five to week twenty-seven before the index date. The twenty-six-week skip was inserted to ensure that symptoms of undiagnosed CML did not influence the evaluation of work ability before diagnosis.

Cohabiting status and the highest educational level were categorized according to the status the year before index date. The educational level was grouped in four based on the ISCED11 level (ISCED 0–2, 3, 5–6, and 7–8) retrieved from the Danish Education Registries [22,23].

Equalized income (corresponding to the total disposable income for the household adjusted to the size of the household by the OECD-modified equivalence scale) was obtained the year before the index date from The Income Statistics Register [24,25]. The equalized income was grouped into four based on the patients’ and matched comparators’ age and their year of inclusion. The method is described in detail elsewhere [26].

### 2.3. Statistical Analysis

Baseline characteristics for both CML patients and matched comparators were presented as frequencies and percentages for categorical variables and as medians and interquartile ranges (IQRs) for continuous variables. Overall survival was computed using the Kaplan–Meier estimator and differences between patients with CML and matched comparators were tested using a log-rank test. Median follow-up was calculated using the reverse Kaplan–Meier estimator.

In the primary analysis, the distribution of individuals in relation to work ability status was examined at each week following the index date. Fischer’s exact test was used to test whether the proportion of working, sick leave, flexible job, and disability pension differed between patients with CML and matched comparators at 1, 3, 5, and 10 years after the index date. In this analysis, participants were removed from the risk set when censored (31 December 2021 or emigration) or at the time of death.

In the secondary analysis, participants were followed from the index date until the work ability event (date of either disability pension or flexible job), competing event (eventual future CML diagnosis for matched comparators, death, early retirement pension, or date of entitlement to retirement pension), or censoring (31 December 2021 or emigration), whichever occurred first. Age at entitlement to retirement pension depends on birth year and is listed in Supplemental Table S2. The cumulative risks of disability pension and flexible job were calculated using the Aalen–Johansen estimator, and differences between patients and matched comparators were tested using Gray’s test [27,28]. For the cumulative risk of requiring flexible job, disability pension was also treated as a competing event. Also, crude cause-specific hazard ratios (HRs) of disability pension and flexible job were calculated using Cox proportional hazards regression. Visual inspection of the Schoenfeld residuals was used to confirm the assumption of proportional hazards.

An exploratory analysis investigating the factors associated with the risk of flexible job and disability pension among patients with CML at 5 years after diagnosis was performed by the calculation of risk differences (RDs) in subgroups of patients with CML using a pseudo-observation approach. Both crude RDs and RDs adjusted for sex and age (25–36, 37–48, and 49–60 years) were presented.

All analyses were performed in SAS version 9.4 (SAS Institute Inc, Gary, NC, USA) and R version 4.0.3 (R foundation for Statistical Computing, Vienna, Austria).

## 3. Results

### 3.1. Patients and Matched Comparators

This study included 489 patients with CML and 2445 matched comparators with a median age of 46 years. The male sex was dominant (59.5%). Patients with CML were more prone to receive sick leave compensation six months before diagnosis than matched comparators (6.1% vs. 3.4%, Table 1). Patients with CML and matched comparators were followed for a median of 8.7 years (95% confidence interval [CI]: 7.9;9.6 years) and 9.3 years (95% CI: 9.0;9.6, Supplemental Table S3), respectively. Overall survival differed between patients and matched comparators (*p* < 0.0001, Supplemental Figure S1).

### 3.2. Work Ability

**Figure 1:**
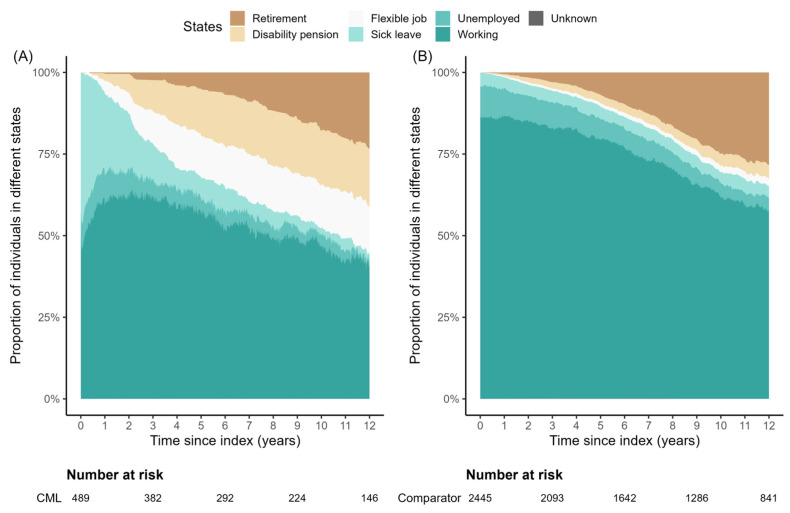
Work ability after index date (corresponding to date of diagnosis) for (**A**) patients with CML and (**B**) matched comparators. Retirement includes both retirement pension and early retirement pension. Number at risk included patients and matched comparators that were alive and not censored due to end of study or emigration. Abbreviations: CML, chronic myeloid leukemia.

The work ability after the index date for patients and matched comparators is shown in Figure 1. At index date, only 50.1% of the patients were working, as opposed to 86.3% of the matched comparators (*p* < 0.001). The proportion of patients in work also differed from matched comparators at 1, 3, 5, and 10 years after the index date (*p* < 0.001). Furthermore, the proportion of sick leave differed between patients and matched comparators at index date and 1, 3, and 5 years after the index date, with a higher proportion of patients needing sick leave compared to matched comparators (*p* < 0.001–0.024). Finally, the proportion of flexible jobs and disability pensions differed at 1, 3, 5, and 10 years after the index date, with a higher need for permanent disability compensation among patients (*p* < 0.001, Table 2).

### 3.3. Risk of Flexible Job and Disability Pension

**Figure 2:**
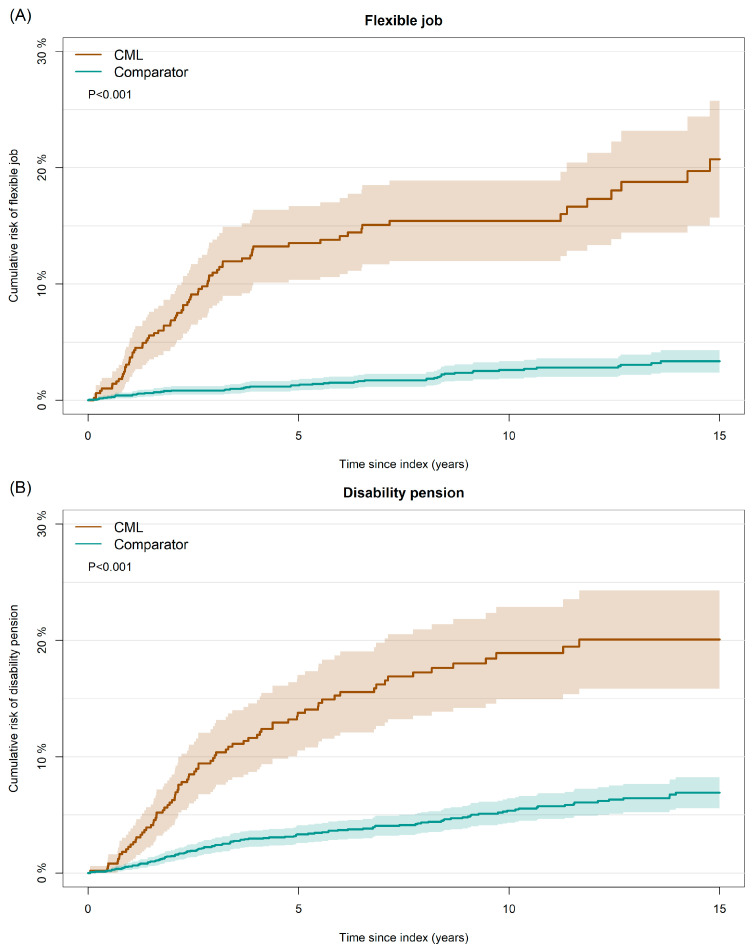
Cumulative risk of (**A**) flexible job and (**B**) disability pension among patients with CML and matched comparators. Abbreviations: CML, chronic myeloid leukemia.

Patients diagnosed with CML had a higher hazard rate of being granted both flexible job and disability pension compared to matched comparators, with HR 8.7 (95% CI: 6.1;12.2, *p* < 0.001) and HR 3.7 (95% CI: 2.8;4.9, *p* < 0.001), respectively. The same was evident for the cumulative risk of flexible job and disability pension (*p* < 0.001, Figure 2A,B). The 5-year cumulative risk of flexible job was 13.5% (95% CI: 10.3;16.7) for patients and 1.3% (95% CI: 0.8;1.8) for matched comparators. The 5-year cumulative risk of disability pension was 13.8% (95% CI: 10.5;17.0) for patients and 3.4% (95% CI: 2.6;4.1) for matched comparators.

Five years after diagnosis, the exploratory analysis identified the female sex and long-term sick leave before the index date as significant risk factors for flexible job. The risk of disability pension five years after diagnosis was significantly associated with the female sex, higher age, living alone, lower income, and long-term sick leave before the index date. Contrary, the achievement of the second highest educational level was associated with a decreased risk of disability pension (Table 3).

## 4. Discussion

This nationwide cohort study investigated work ability in patients diagnosed with CML compared to the Danish general population. This study revealed that patients with CML have increased rates of both temporary and permanent reduced work ability, with cause-specific HRs of 8.7 and 3.7 for flexible job and disability pension, respectively.

Furthermore, a statistically significantly higher percentage of patients with CML needed sick leave at the time of diagnosis compared to their matched comparators. Likewise, this study revealed a tendency towards a higher percentage of patients receiving sick leave compensation six months before diagnosis. Even though some patients are asymptomatic at the time of diagnosis, other patients experience symptoms like fatigue, fever, night sweat, pain in the bones and spleen, and weight loss. Among those, especially fatigue and pain can have an impact on work ability. The results of the present study indicate that the symptoms could have an influence on everyday life before a diagnosis is established. Reduced capacity to work at the time of diagnosis was also described in a study by Svingel et al. [29]. The study examined labor market affiliation among patients with Philadelphia-negative myeloproliferative neoplasms (MPNs) and found patients with MPN to be less likely to work (patients with essential thrombocythemia, polycythemia vera, and unclassifiable MPN) and more likely to receive sick leave compensation (patients with all subtypes) compared to sex- and age-matched comparators.

The proportion of patients with CML that were able to work increased within the first year after diagnosis, probably reflecting a reduction in symptoms after treatment initiation. Since the early 2000s, the standard treatment for CML has been TKIs. TKIs are orally administrated drugs, which is much more compatible with an active work life than traditional chemotherapy [3]. However, studies have found that only 56.9% of patients are strictly adherent to the treatment, and more than half of patients experience adverse reactions, including, e.g., fatigue, musculoskeletal pain, nausea, vomiting, and diarrhea [30,31]. This could contribute to the reduced work ability among patients with CML in the present study. The proportion of patients in need of sick leave compensation was increased up to 5 years after diagnosis. This could reflect the fact that finding the right treatment takes time due to, e.g., resistance mechanisms and adverse effects [32]. Some patients may need sick leave compensation during this process. This is supported by a study by Conte et al., which showed that the proportion of patients in need of sick leave increased after the initiation of TKI treatment [8].

Short-term sick leave (comprising 14–30 days within the study period) is, according to the Danish legislation, paid by the employer and therefore not registered in the DREAM register unless a special agreement has been entered. This agreement can be entered by employees with chronic diseases, and it gives the employer full compensation from the state from the first day of an employee’s absence. This absence is registered as sick leave in the DREAM register. This could also be an explanation for the higher sick leave among patients with CML compared to the matched comparators, as patients with CML are more likely to have entered this agreement. However, the proportions of sick leave after ten years were not significantly different between patients with CML and the matched comparators, which argues against this being the only explanation for the increased rates of sick leave.

The risk of permanent loss of work ability (flexible job and disability pension) was increased among patients with CML compared to matched comparators. In previous studies, this was also observed among patients with other hematological diseases, like lymphoma, multiple myeloma, and acute leukemia, but not among patients with MPNs [5,6,26,29,33]. However, the median age in the study by Svingel et al. was equal to or exceeded the age of retirement pension, hindering the granting of flexible job or disability pension [29]. The increased risk of flexible job corresponds to an increased need for individual adjusted work functions, e.g., reduced work time and modified work functions. This is similar to a study by De Barros et al., who found that within the first year after a diagnosis of CML, two out of three patients needed work modifications, with reduced working hours being the most required [7].

Within the present study, the cumulative risk of requiring flexible job increased up to four years after diagnosis, and then after a stable period, the need increased again approximately eleven years after diagnosis. However, the risk of disability pension stabilized approximately ten years after diagnosis. In 2013, the Danish legislation on disability pension was modified to increase work maintenance, which led to fewer patients with cancer being granted disability pension [34]. For patients diagnosed early in our study period, this could have led to more patients being granted flexible job instead of disability pension.

There was a trend towards a larger proportion of matched comparators being retired >5 years after index date. Retirement included early retirement, a special retirement scheme that is possible for citizens that have paid into the scheme and who have not been granted either flexible job or disability pension. It is a possibility that matched comparators were more prone to retire using this scheme than patients with CML. Likewise, as the cumulative incidence of both flexible job and disability pension is significantly increased among patients with CML, the possibility of using the early retirement scheme is consequently reduced.

The loss of work ability is multifactorial and may be affected by both physical and physiological disorders. Being diagnosed with a chronic cancer disease may affect many aspects of life. A study by Øvlisen et al. found that patients with chronic indolent lymphoma disease were more likely to receive long-term antidepressants than patients with aggressive lymphoma [35]. This indicates that living with a chronic disease can result in other complications that may also affect the ability to work.

Within the exploratory analysis, the 5-year risks of requiring flexible job and disability pension were both associated with the female sex and a higher need for sick leave within the year before diagnosis. For disability pension alone, a higher age, living alone, and lower income also increased the risk. These factors are well-known risk factors for disability pension [36]. We found no certain correlation between the risk of a reduced work capacity and educational level, which is another known risk factor. This could be caused by the low number of patients, especially in the highest education level.

This study has several strengths, including the use of high-quality registries with almost complete data capture. As patients with CML were identified in both the Danish National Pathology Registry and the Danish National Chronic Myeloid Neoplasia Registry, the latter being mandatory for all hematological departments in Denmark to report to, a high completeness of identification of patients with CML must be anticipated. Furthermore, we were able to combine information across registries and hence assure almost complete follow-up.

The drawbacks include a lack of information on why patients and matched comparators were unable to work. Patients with CML can lose work ability for many other reasons. Within the present study, patients with CML were more likely to have long-term sick leave before diagnosis. This is a known risk factor for work disability and could have increased the risk of disability within the group of patients. Furthermore, we did not match patients and comparators on level of education, a known predictor for loss of work ability. However, the levels were reasonably distributed, even though there was a trend towards a lower level of education among patients with CML. On the other hand, the equalized income for CML patients tended to be higher. These two are often correlated, and the little differences must again be related to the small subgroups. Finally, the registries used within this study did not include information on the quality of life. As work ability is highly correlated to quality of life, this information would have been relevant to include if available.

## 5. Conclusions

Compared to the general population, patients with CML have a reduced work ability, demonstrated by a significantly higher risk of sick leave, flexible jobs, and disability pensions following a disease diagnosis. Flexible jobs and disability pensions are granted to patients with permanent loss of work ability, meaning that patients with CML, even though often well treated for their CML, could have a reduced quality of life due to the loss of work ability. Further studies are needed to address what causes the loss of work ability.

## Figures and Tables

**Table 1:** Baseline characteristics for patients with CML and matched comparators. Abbreviations: CML, chronic myeloid leukemia; CCI, Charlson comorbidity index; ISCED, International Standard Classification of Education.

	Patients with CML (*n* = 489)	Matched Comparators (*n* = 2445)
**Age, median (IQR)**	46 (39–54)	46 (38–54)
**Age group, *n* (%)**		
25–36 years	101 (20.7%)	503 (20.6%)
37–48 years	181 (37.0%)	902 (36.9%)
49–60 years	207 (42.3%)	1040 (42.5%)
**Sex, *n* (%)**		
Male	291 (59.5%)	1455 (59.5%)
Female	198 (40.5%)	990 (40.5%)
**CCI prior to diagnosis, *n* (%)**		
0	406 (83.0%)	2030 (83.0%)
1	50 (10.2%)	250 (10.2%)
≥2	33 (6.7%)	165 (6.7%)
**Cohabiting status, *n* (%)**		
Living alone	118 (24.1%)	677 (27.7%)
Living with partner	366 (74.8%)	1744 (71.3%)
Unknown	5 (1.0%)	24 (1.0%)
**Education level (ISCED), *n* (%)**		
ISCED 0–2	98 (20.0%)	436 (17.8%)
ISCED 3	232 (47.4%)	1126 (46.1%)
ISCED 5–6	97 (19.8%)	595 (24.3%)
ISCED 7–8	48 (9.8%)	225 (9.2%)
Unknown	14 (2.9%)	63 (2.6%)
**Equalized income, quartiles, *n* (%)**		
Lowest	83 (17.0%)	493 (20.2%)
Second lowest	136 (27.8%)	577 (23.6%)
Second highest	127 (26.0%)	683 (27.9%)
Highest	138 (28.2%)	668 (27.3%)
Unknown	5 (1.0%)	24 (1.0%)
**Employment status ^1^, *n* (%)**		
Working	410 (83.8%)	2149 (87.9%)
Unemployed	42 (8.6%)	197 (8.1%)
Sick leave	30 (6.1%)	83 (3.4%)
Unclassified	7 (1.4%)	16 (0.7%)
**Long-term sickness ^2^, *n* (%)**		
Yes	59 (12.1%)	198 (8.1%)
No	430 (87.9%)	2247 (91.9%)
**Year of diagnosis, *n* (%)**		
2002–2007	139 (28.4%)	-
2008–2013	156 (31.9%)	-
2014–2020	194 (39.7%)	-

^1^ Employment status is calculated over nine weeks from week thirty-five to week twenty-seven before index date. ^2^ Four or more consecutive weeks within week twenty-seven to seventy-eight before diagnosis or similar date for comparators.

**Table 2:** Proportion of patients with CML and matched comparators working or receiving sick leave, flexible job compensation, or disability pension. Abbreviations: CML, chronic myeloid leukemia.

Time Since Index (Years)	Working State	Patients with CML (%)	Matched Comparators (%)	*p*-Value
0	Working	50.1	86.2	<0.001
	Sick leave ^1^	42.3	4.3	<0.001
	Flexible job	0	0	-
	Disability pension	0	0	-
1	Working	59.2	86.5	<0.001
	Sick leave ^1^	25.5	3.3	<0.001
	Flexible job	3.9	0.4	<0.001
	Disability pension	2.2	0.5	0.002
3	Working	61.8	83.0	<0.001
	Sick leave ^1^	11.3	3.3	<0.001
	Flexible job	9.9	0.7	<0.001
	Disability pension	9.4	1.9	<0.001
5	Working	56.8	79.3	<0.001
	Sick leave ^1^	6.8	3.8	0.024
	Flexible job	12.7	1.0	<0.001
	Disability pension	14.3	2.6	<0.001
10	Working	46.7	61.8	<0.001
	Sick leave ^1^	2.0	3.4	0.505
	Flexible job	13.2	1.9	<0.001
	Disability pension	16.8	3.7	<0.001

^1^ Sick leave only includes temporary sick leave payments.

**Table 3:** Risk of disability pension and flexible job and crude and adjusted risk difference between subgroups at five years after index date. Abbreviations: RD, risk difference; ISCED, International Standard Classification of Education; CCI, Charlson comorbidity index.

	Flexible Job			Disability Pension		
	Risk (%)	RD (Crude)	RD (Adjusted) ^1^	Risk (%)	RD (Crude)	RD (Adjusted) ^1^
**Sex**						
Male	7.8 (4.6;11.1)	0		9.7 (6.1;13.4)	0	
Female	21.9 (15.8;27.9)	14.0 (7.2;20.9) *		19.7 (13.9;25.5)	10.0 (3.1;16.9) *	
**Age at diagnosis**						
25–36 years	13.9 (6.9;21.0)	0		4.4 (0.2;8.7)	0	
37–48 years	13.3 (8.2;18.4)	−0.7 (−9.6;8.2)		12.9 (7.8;17.9)	8.6 (1.7;15.4) *	
49–60 years	13.6 (8.6;18.6)	−0.4 (−9.0;8.3)		19.5 (13.6;25.4)	14.9 (7.8;22.1) *	
**Year of diagnosis**						
2002–2007	16.8 (10.5;23.0)	0	0	17.5 (11.2;23.9)	0	0
2008–2013	13.5 (8.1;18.8)	−3.0 (−11.9;5.8)	−3.1 (−11.7;5.5)	12.8 (7.6;18.1)	−5.3 (−14.4;3.8)	−5.9 (−14.8;3.0)
2014–2020	11.6 (6.2;17.1)	−5.2 (−13.1;2.7)	−5.4 (−13.2;2.4)	12.5 (6.6;18.5)	−5.8 (−14.1;2.5)	−6.4 (−14.7;1.9)
**Cohabiting status**						
Living alone	8.8 (3.3;14.2)	0	0	18.2 (10.7;25.6)	0	0
Living with partner	14.9 (11.1;18.7)	6.1 (−0.5;12.7)	5.8 (−1.1;12.8)	12.6 (9.0;16.1)	−5.5 (−13.7;2.7)	−8.3 (−16.4;−0.2) *
**Education level**						
ISCED 0–2	17.0 (9.4;24.6)	0	0	21.3 (12.7;29.8)	0	0
ISCED 3	13.3 (8.8;17.9)	−3.8 (−12.9;5.3)	−3.1 (−11.9;5.7)	12.7 (8.2;17.2)	−8.8 (−18.7;1.2)	−8.1 (−17.9;1.7)
ISCED 5–6	12.9 (5.7;20.1)	−4.3 (−15.0;6.3)	−5.3 (−15.4;4.9)	10.5 (4.0;17.0)	−10.9 (−21.9;0.1)	−12.1 (−22.8;−1.5) *
ISCED 7–8	7.2 (0.0;15.1)	−9.9 (−21.1;1.4)	−9.7 (−20.7;1.3)	11.1 (1.9;20.4)	−10.3 (−23.2;2.7)	−8.8 (−21.5;3.9)
**Equalized income**						
Lowest	15.3 (7.3;23.3)	0	0	24.6 (14.9;34.3)	0	0
Second lowest	18.4 (11.5;25.2)	3.0 (−7.8;13.8)	3.2 (−7.6;14.0)	18.6 (11.7;25.5)	−6.6 (−18.9;5.8)	−12.3 (−24.0;−0.7) *
Second highest	12.4 (6.5;18.3)	−3.0 (−13.2;7.2)	−3.1 (−13.4;7.2)	10.4 (4.8;16.1)	−14.7 (−26.4;−3.1) *	−18.6 (−29.3;−8.0) *
Highest	8.1 (3.2;12.9)	−7.3 (−16.9;2.3)	−6.6 (−16.5;3.3)	5.5 (1.5;9.6)	−19.6 (−30.6;−8.6) *	−22.1 (−32.4;−11.9) *
**Long-term sickness ^2^**						
No sick leave	11.5 (8.3;14.6)	0	0	11.7 (8.5;15.0)	0	0
Sick leave	28.5 (16.6;40.3)	16.9 (4.5;29.4) *	15.4 (2.9;27.9) *	28.6 (16.6;40.6)	17.1 (4.6;29.5) *	15.0 (2.7;27.4) *
**CCI prior to diagnosis**						
0	13.5 (10.0;17.0)	0	0	12.1 (8.7;15.4)	0	0
1	15.8 (5.0;26.6)	2.2 (−8.8;13.1)	2.5 (−8.9;13.9)	22.7 (10.2;35.2)	10.4 (−2.1;23.0)	9.2 (−2.9;21.3)
≥2	10.1 (0.0;21.1)	−3.1 (−14.5;8.3)	−3.4 (−15.4;8.7)	24.2 (6.7;41.8)	10.1 (−5.9;26.1)	7.2 (−8.4;22.7)

^1^ RD (adjusted) was adjusted for sex and age (25–36, 37–48, and 49–60 years). ^2^ Four or more consecutive weeks within week twenty-seven to seventy-eight before diagnosis. * significant.

## Data Availability

This study was performed on a secured remote research platform hosted at Statistics Denmark without the possibility to share data beside figures and tables outside the platform. Hence, no data sharing is possible.

## Supplementary Materials

The following supporting information can be downloaded at: https://www.mdpi.com/article/10.3390/cancers17091585/s1, Figure S1: Overall survival for patients with CML and matched comparators; Table S1: Grouping of social payments and work according to DREAM codes; Table S2: Age at entitlement to retirement pension according to year of birth; Table S3: Follow-up time categorized by endpoint.

## Abbreviations

The following abbreviations are used in this manuscript:

CML	chronic myeloid leukemia
TKI	Tyrosine Kinase Inhibitor
IQR	Interquartile Range
CRS	The Danish Civil Registration System
CCI	Charlson comorbidity index
RD	Risk difference
MPN	Philadelphia-negative myeloproliferative neoplasm
