# Supplementary material for: Work Ability in Patients with Chronic Myeloid Leukemia: A Danish Nationwide Cohort Study
Source: Cancers (Basel). 2025 May 7;17(9):1585. doi: 10.3390/cancers17091585 (PMC12072068; doi:10.3390/cancers17091585)
Supplement: Supplementary file 1 [file cancers-17-01585-s001.zip › cancers-3576142-supplementary.pdf]

Supplemental Material

Table S1. Grouping of social payments and work according to DREAM codes.

Work status	Including	Code in DREAM
Working	Not receiver of any public transfer payment	Weeks without a code in DREAM
	State education grant and other education	413, 521, 522, 651, 652, 661
	Parental leave	881
	Leave of absence	121-126, 412
Unemployed	Unemployment compensation	111-115, 211-219, 231-232, 299
	Social security benefits	130-169, 511, 611, 700-739
	Rehabilitation benefits	750-768
Sick leave	Sick pay	870-878, 890, 892-899
	Vocational rehabilitation program	810-818
Flexible job		622, 740-748, 771-774
Disability pension		781-785
Early retirement pension		621
Retirement pension		996, 998
Unclassified	Living outside Denmark	997

Table S2. Age at entitlement to retirement pension according to year of birth.

**Birth year**	**Age at entitlement to retirement pension**
1942-1953	65 years
1954 (first half of the year)	65.5 years
1954 (second half of the year)	66 years
1955 (first half of the year)	66.5 years
1955 (second half of the year) - 1995	≥ 67 years<br>*This part of the cohort does not reach retirement age within the study period.*

Table S3. Follow-up time categorized by endpoint.

	Median (95% CI)
**Risk of disability pension**	
Patients with CML	9.0 (8.0;9.9)
Matched comparators	9.3 (9.0;9.6)
**Risk of flexible job**	
Patients with CML	8.7 (7.9;9.6)
Matched comparators	9.3 (9.0;9.6)

Abbreviations: CML, Chronic Myeloid Leukemia

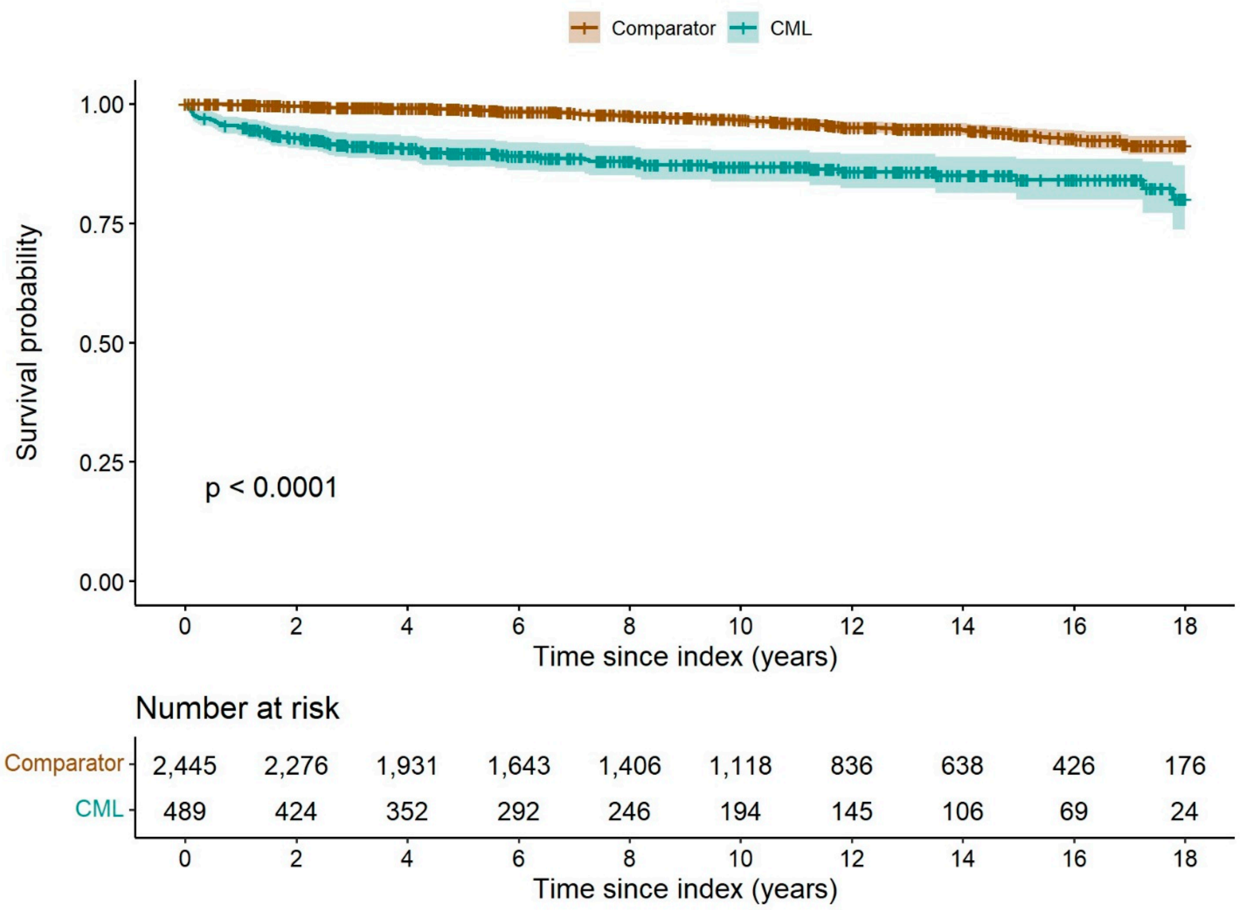

Abbreviations: CML, Chronic Myeloid Leukemia

Figure S1. Overall survival for patients with CML and matched comparators.
